# Electronic Structure of Nitrobenzene: A Benchmark Example of the Accuracy of the Multi-State CASPT2 Theory

**DOI:** 10.1021/acs.jpca.1c04595

**Published:** 2021-10-22

**Authors:** Juan Soto, Manuel Algarra

**Affiliations:** † Department of Physical Chemistry, Faculty of Science, University of Málaga, Málaga 29071, Spain; ‡ Department of Inorganic Chemistry, Faculty of Science, University of Málaga, Málaga 29071, Spain

## Abstract

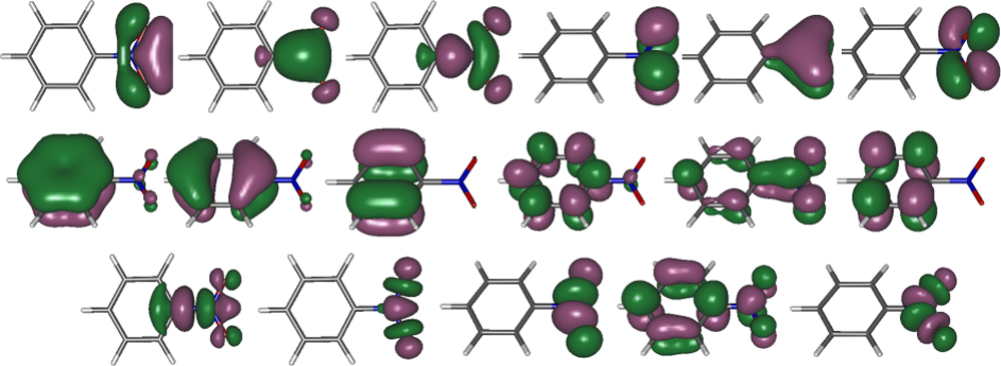

The electronic structure of nitrobenzene (C_6_H_5_NO_2_) has been studied by means of the complete active space self-consistent field (CASSCF) and multi-state second-order perturbation (MS-CASPT2) methods. To this end, an active space of 20 electrons distributed in 17 orbitals has been selected to construct the reference wave function. In this work, we have calculated the vertical excitation energies and the energy barrier for the dissociation of the molecule on the ground state into phenyl and nitrogen dioxide. After applying the corresponding vibrational corrections to the electronic energies, it is demonstrated that the MS-CASPT2//CASSCF values obtained in this work yield an excellent agreement between calculated and experimental data. In addition, other active spaces of lower size have been applied to the system in order to check the active space dependence in the results.

## Introduction

Although nitrobenzene (C_6_H_5_NO_2_) is the smallest molecule of the nitroaromatic compound family, it presents rich chemistry. Its thermal and photochemical decompositions are important in several different areas such as combustion, decomposition of energetic materials, or atmospheric chemistry.^1−3^ For this reason, the photophysics, photochemical, and thermal dissociation reactions of nitrobenzene have been studied by many different groups, both experimentally^1,4−11^ and theoretically.^11−17^ Concerning the theoretical studies, a wide variety of quantum chemical methods have been employed to elucidate the excitation and decomposition processes.^11−17^ Given that nitrobenzene is a strongly correlated system, the complete active space self-consistent field (CASSCF) method is one of the most adequate approaches for studying such a compound.^18−21^ Unfortunately, due to exponential growth in the computational cost,^22^ the application of exact CASSCF is limited to small size active spaces, whose limit is approximately 20 electrons distributed in 20 orbitals; only when massive parallelization was implemented,^23^ it was possible to enlarge the active space to 22 electrons distributed in 22 orbitals. To overcome this drawback, new approaches and methods are being developed with the objective of enlarging the treatable active spaces or select the optimal minimum of active orbitals.^18−25^ On the other hand, until now, the general tendency was to select by hand the minimal active space in accordance with the chemical problem under study. However, this methodology has serious inconveniences when orbitals are strongly correlated and could lead to inexact results or erroneous conclusions. In this context, nitro-derivatives are paradigmatic examples.^26−32^ In particular, nitrobenzene is at the limit of the CASSCF capabilities because it demands an active space of 20 electrons distributed in 17 orbitals.^25−31^ For this reason, the application of the CASSCF method to this system with an active space of this size is a challenging task due to the huge number of electrons and orbitals that has to be included. However, the objective of this work is to treat nitrobenzene with such a large active space. We will demonstrate in this work that the multi-state second-order perturbation (MS-CASPT2)/CASSCF method yields excellent predictions compared with experimental data, that is, vibrationally corrected vertical excitations and dissociation energies.

## Theoretical Methods

The CASSCF,^33−39^ the MS-CASPT2,^40,41^ and multiconfiguration pair-density functional theory (MC-PDFT)^42−50^ methods have been applied as implemented in MOLCAS 8.4.^51,52^ The MC-PDFT density method has been used as a computationally economical alternative to MS-CASPT2, which is a type of density functional theory that combines Kohn–Sham density functional theory and multiconfiguration wave function theories by using the electron density and pair density from a previous multiconfigurational calculation. Thus, any multiconfigurational method that is able to provide one- and two-electron reduced density matrices can be used as a starting point to applied MC-PDFT; typically, CASSCF reference wave functions are the most commonly used, and for this reason, it is subjected to the same computational limitations as such a method. MS-CASPT2 energies have been calculated with the application of an imaginary shift set to 0.1 in order to avoid the inclusion of intruder states in the calculations. Equally, the IPEA empirical correction has been fixed at the standard value (0.25) in all of the calculations. CASSCF applied with the state average approximation is noted as SA*n*-CASSCF where *n* refers to the number of states of a given symmetry species. The so-called atomic natural orbital (ANO)-RCC basis sets,^53−55^ that is, extended relativistic basis sets of the ANO-type, have been used in the multiconfigurational calculations of this work by applying the following contraction scheme: (C,N,O)[4s3p2d1f]/(H)[3s2p1d].

The one-dimensional potential energy surfaces for the dissociation reaction of nitrobenzene are built with an interpolation method^56−60^ using the full space of nonredundant internal coordinates, which provides an accurate one-dimensional representation of the potential energy surfaces in the space spanned by a given set of internal coordinates. To achieve this end, first, a common set of 3N-6 internal coordinates is defined for the target geometries, the reactant (**R**_1_), and the products (**R**_2_). For reactions in which bond-breaking is involved, our experience^61−65^ shows that an internuclear distance of ∼4.7 Å of the bond to be broken (C–N distance for dissociation of nitrobenzene into phenyl and NO_2_) is adequate to reach the asymptotic limit of the potential energy surface (PES)of interest. Second, the difference between **R**_2_ and **R**_1_ yields an interpolation vector (Δ**R**) that connects reactants and products. Third, Δ**R** is divided by *n* (an entire number at the choice of the user). Each of the divisions constitutes what we will call a *step*. In consequence, each step *m* corresponds to a nuclear configuration given by **R**_m_ = **R**_1_ + (*m*/*n*)Δ**R**. Because we use internal coordinates (internuclear distances, valence bonds, and dihedral angles), we cannot give a unique unit for the reaction coordinate. Therefore, in what follows, we will indicate them as *arbitrary units*. Linear interpolations in internal coordinates present two favorable characteristics: (i) They are less demanding computationally than a scan with the relaxation of geometry and (ii) all the points along the interpolation vector (reaction coordinate) are disposed in a straight line, which is not true for the scanning of the potential energy surfaces with geometry relaxation.

To finish this section, the geometries and molecular orbitals of the chemical species have been analyzed with the Gabedit,^66^ Molden,^67^ and MacMolplt programs.^68^ The charge distribution has been analyzed with the LoProp method,^69^ which has the advantage to avoid the dependence of the computed atomic charges with the basis sets. The method requires a subdivision of the atomic basis sets for each atom of the molecule into occupied and virtual basis functions, which will be orthogonalized to yield a localized orthonormal basis set.

### Selection of the Active Space

**Figure 1:**
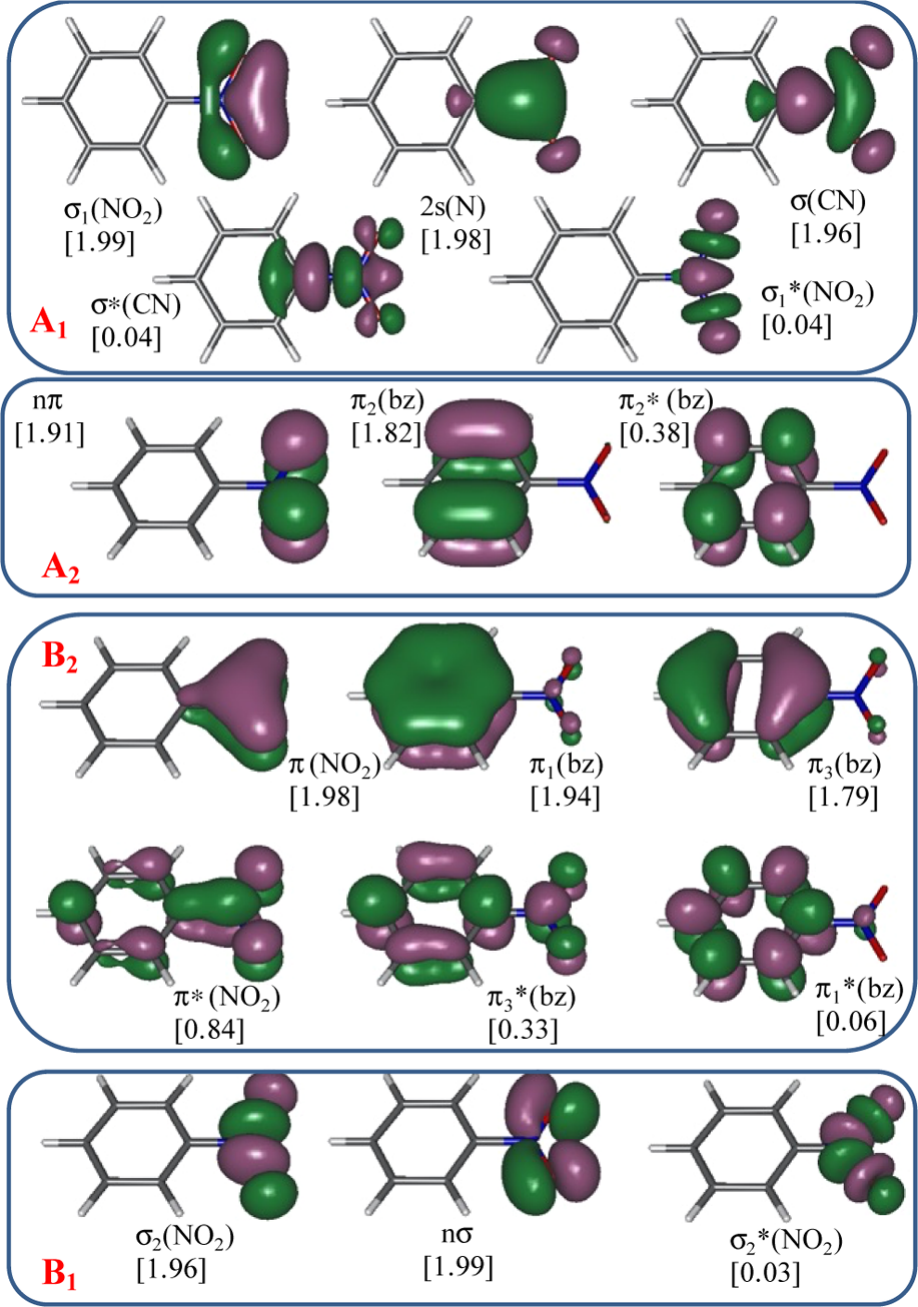
CASSCF/ANO-RCC natural orbitals included in the active space (20e and 17o) of the ground state CASSCF optimized geometry of nitrobenzene. In square brackets: occupation numbers.

Given that we are mainly interested in the dissociation of nitrobenzene into phenyl and NO_2_ in the ground and excited states, as well as the study of singlet and triplet excitations, the active space must include 20 electrons distributed in 17 orbitals. The selection of the active space of the molecule is straightforward in accordance with the two fragments that compose the molecule (NO_2_ and phenyl). These arise as follows: the NO_2_ moiety demands an active space of 13 electrons distributed in 10 orbitals,^26−32^ and other selection of the active orbitals will lead to symmetry breaking of the wave function of this radical. In addition, the phenyl fragment requires an active space of seven electrons in seven orbitals,^62^ six electrons, and six orbitals corresponding to the π-system plus the singly occupied σ-orbital involved in the C–N bond. Thus, with this active space, we can adequately treat both the inter- and intra-electronic transitions between the two fragments plus the C–N bond breaking, avoiding misunderstanding results.^70−72^Figure 1 shows the state-average CASSCF orbitals of nitrobenzene included in the active space along with the character assigned to them. They were optimized under *C*_2v_ restriction and correspond to the A_1_ symmetry species.

## Results and Discussion

### Energetics of the Singlet and Triplet States at the Franck–Condon Geometry

The gas phase absorption spectrum of nitrobenzene shows two very weak bands with maxima at 350 and 280 nm together with two strong bands with maxima at 240 and 193 nm.^1,5−8^ Concerning theoretical studies, vertical excitation energies of the singlet and triplet excited states of nitrobenzene have been widely studied by other authors^10,13−17^ at the CASPT2//CASSCF level. However, given that they were mainly interested in other physical and chemical aspects of nitrobenzene, for example, reactivity in the excited states, they were obliged to work with a smaller active space than is used in this work. In this work, the MS-CASPT2 approximation for calculating the singlet and triplet vertical excitation energies has been applied by taking a large active space to build the reference wave function (Table 1). The weights of the reference CASSCF functions were ≥0.70 for all the singlet and triplet states. Given that vertical excitations are highly dependent on the molecular parameters and it is well known that MP2 predicts molecular geometries in better agreement with the experimental results in comparison with the data obtained from the CASSCF approach, we have calculated such vertical excitations on top of the C_2v_ MP2 geometry. Vertical excitations at the CASSCF geometry are listed in Table S1. The experimental and calculated geometrical parameters are given in Table S2. At this point, it is convenient to highlight that the average torsional angle of the nitro group determined by gas-phase electron diffraction experiments^73^ amounts to 13.3 ± 1.4°; however, this average (dynamic) value is not directly comparable with the equilibrium (static) parameter because two factors absent at equilibrium operate on the average parameter: (i) the torsional vibration of the nitro moiety and (ii) the very low barrier to rotation of such a moiety. Thus, the results reported in Table 1 show a very good agreement between calculated and observed transitions when vibrational corrections are applied (vibrational energy correction in Table 2). Such corrections are done by taking the zero-point energies of the ground and the excited state of each transition. It must be remarked that these corrections do not correspond to the 0–0 transitions of each transition. In contrast, the vibrational energy of each excited state is calculated at the Franck–Condon point after rotation of the corresponding Hessian matrix, that is, the gradient and the rotational and translational normal modes are projected out of the respective excited Hessian matrix before computing the corresponding vibrational frequencies,^74−76^ which were calculated with the CAM-B3LYP functional in conjunction with the def2-TZVPP basis sets. Fortunately, most of the observed transitions in the experiments correspond to single excitations, which allows for the application of the standard density functional theory to support the vibrational correction. In addition, our calculations correctly predict the intensity of the bands, except the one localized at the shortest wavelength. The reason for this behavior of the calculations probably lies in the nature of the band, which is a transition where the breathing vibration of the benzenic ring is involved.^1^ Thus, given that the C–C sigma orbitals of the benzenic ring were not included in the active space, this transition is not well described.

**Table 1:** Vertical Excitation Energies in eV of the Singlet and Triplet States of Nitrobenzene (*C*_2v_, MS-CASPT2).^a^^,^^b^

state	Δ*E*	f_OSC_^c^	configuration^d^	*W*^e^	Δ*Q*^f^
2^1^A_1_	**5.11**	2.94–01	[π_3_(bz)]^1^[π*(NO_2_)]^1^	72	–0.32
3^1^A_1_	7.60	1.06–02	[π_1_(bz)]^1^[π*(NO_2_)]^1^	33	–0.21
			[π_3_(bz)]^0^[π*(NO_2_)]^2^	15	
1^1^A_2_	3.83	<1.0–05	[nσ]^1^[π*(NO_2_)]^1^	68	+0.14
2^1^A_2_	7.00	<1.0–05	[nσ]^1^[π_3_(bz)]^1^[π*(NO_2_)]^2^	27	+0.18
			[nσ]^1^[π_3_*(bz)]^1^	32	
3^1^A_2_	7.36	<1.0–05	[σ_1_(NO_2_)]^1^[σ_2_*(NO_2_)]^1^	55	+0.35
1^1^B_2_	4.30	1.01–04	[σ_1_(NO_2_)]^1^[π*(NO_2_)]^1^	66	+0.08
2^1^B_2_	7.02	1.13–05	[nσ]^1^[π_2_*(bz)]^1^	49	+0.29
			[nσ]^1^[π_2_(bz)]^1^[π*(NO_2_)]^2^	17	
3^1^B_2_	7.51	3.59–04	[σ_1_(NO_2_)]^1^[π_3_(bz)]^1^[π*(NO_2_)]^2^	21	+0.16
			[σ_1_(NO_2_)]^1^[π_3_*(bz)]^1^	37	
1^1^B_1_	**4.72**	4.83–03	[π_3_(bz)]^1^[π_2_*(bz)]^1^	20	–0.06
			[π_2_(bz)]^1^[π*(NO_2_)]^1^	45	
2^1^B_1_	**5.81**	3.97–02	[nπ]^1^[π*(NO_2_)]^1^	47	+0.15
			[nπ]^1^[π_3_(bz)]^1^[π*(NO_2_)]^2^	12	
3^1^B_1_	**7.04**	8.03–02	[π_3_(bz)]^1^[π_2_*(bz)]^1^	17	–0.18
1^3^A_1_	4.03		[π_3_(bz)]^1^[π*(NO_2_)]^1^	61	–0.03
			[π_2_(bz)]^1^[π_2_*(bz)]^1^	16	
2^3^A_1_	4.75		[π_2_(bz)]^1^[π_2_*(bz)]^1^	59	–0.02
			[π_3_(bz)]^1^[π*(NO_2_)]^1^	15	
3^3^A_1_	6.88		[π_1_(bz)]^1^[π*(NO_2_)]^1^	37	–0.06
			[π_1_(bz)]^1^[π_1_*(bz)]^1^	23	
1^3^A_2_	3.63		[nσ]^1^[π*(NO_2_)]^1^	73	+0.12
1^3^B_2_	4.18		[σ_1_(NO_2_)]^1^[π*(NO_2_)]^1^	67	+0.08
1^3^B_1_	3.53		[nπ]^1^[π*(NO_2_)]^1^	59	+0.12
2^3^B_1_	4.57		[π_2_(bz)]^1^[π*(NO_2_)]^1^	29	–0.05
			[π_3_(bz)]^1^[π_2_*(bz)]^1^	23	
3^3^B_1_	6.03		[π_3_(bz)]^1^[π_2_*(bz)]^1^	42	–0.11
			[π_2_(bz)]^1^[π*(NO_2_)]^1^	22	

a *C*_2v_ MP2/def2-TZVPP optimized geometry.

b SA3-CASSCF reference wave function, IPEA = 0.25. Imaginary shift = 0.1.

c Oscillator strength.

d MS-CASPT2 main electronic configurations of the excited states referred to the ground state configuration.

e Weight of the configuration in %. Only contributions greater than 15% are included.

f Excess of charge on the nitro moiety with respect to the ground state.

**Table 2:** Vibrational Corrected MS-CASPT2 Low-Lying Singlet Vertical Excitations (in eV) of Nitrobenzene at the Ground State C_2v_ geometry and Comparison With Gas-Phase Experimental Results

state	Δ*E*	*VC*^a^	Δ*E*(Corr)^b^	Δ*E*(obs)^c^	refs
1A_2_	3.83	–0.22	**3.61**	**3.65**^d^	(5, 7)
1B_2_	4.30	–0.16	**4.14**		
1B_1_	4.72	–0.23	**4.49**	**4.38–4.43**	(1, 7)
2A_1_	5.11	–0.17	**4.94**	**5.11–5.00**	(1, 7)
2B_1_	5.81	–0.29	**5.53**		
2A_2_	7.00	–0.22	**6.78**		
2B_2_	7.02	–0.27	**6.75**		
3B_1_	7.04	–0.53	**6.51**	**6.42**	(1, 6, 7)
3A_2_	7.36				
3B_2_	7.51				
3A_1_	7.60			**7.56**	(7)

a Vibrational energy correction.

b Corrected excitation energy: Δ*E*(Coor) = Δ*E* + VC.

c Observed absorption peaks.

d Registered in *n*-hexane solution.

For the sake of completeness, the vertical excitation energies of the triplet states are included in Table 1. In accordance with Kröhl et al.,^10^ there are no calculated transitions below 3.0 eV, which agree with the results obtained in the electron energy loss spectra of nitrobenzene. However, the calculated vertical transitions of the triplet states differ notably from the values reported by the cited authors.

### Dissociation of nitrobenzene into phenyl and nitrogen dioxide

Decomposition of simple nitro compounds can occur by a number of possible dissociation pathways. For example, the primary photolysis pathways are^2^
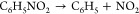
1
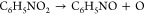
2
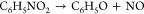
3

**Figure 2:**
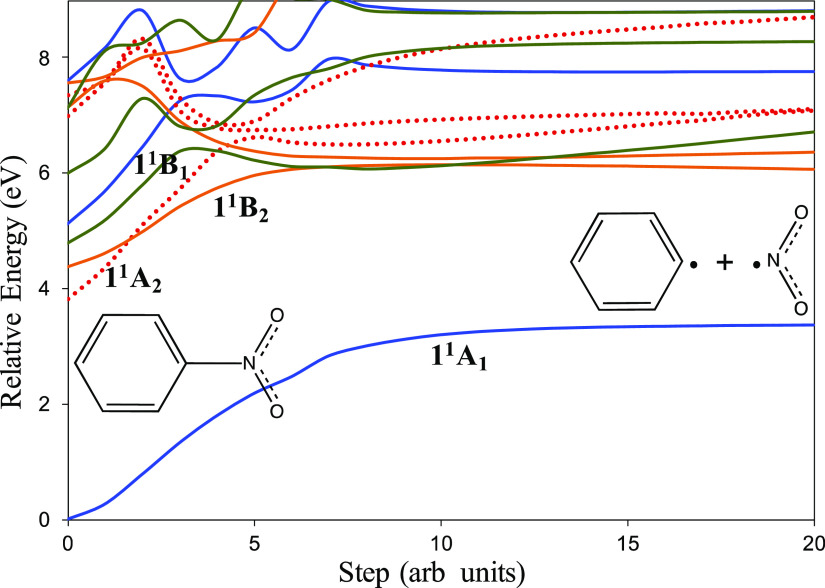
MS-CASPT2/ANO-RCC potential energy curves of the low-lying singlet states of nitrobenzene leading to dissociation into phenyl radical and nitrogen dioxide. Reference wave function: SA3-CASSCF(20e and 17o). A_1_ states (blue lines); A_2_ states (orange dotted lines); B_1_ states (green lines); and B_2_ states (orange solid lines).

Here, we describe the dissociation potential energy surfaces (Figure 2) that would lead to the dissociation of nitrobenzene into phenyl radical and nitrogen dioxide [eq 1], that is, the main coordinate is located on the C–N bond. According to the curves that are represented in Figure 2, the population of the excited states at wavelengths close to the corresponding vertical excitations would not lead to dissociation of the molecule because the energy profiles of such curves are not dissociative. However, it is known^14,15^ that there are several different surface crossings, internal conversions, and intersystem crossings around the Franck–Condon region, for example, it is known that, after excitation into the S_1_ state, nitroaromatic compounds experience ultrafast decaying into the triplet manifold,^14,15^ which competes with internal conversion to the ground state.^14^ To the best of our knowledge, higher internal conversions than S_1_/S_0_ or singlet-triplet intersystem crossings higher than S_0_/T_0_ have not been explored; however, in analogy with other benzene derivatives,^62−65,77,78^ we hypothesize that there will be a multitude of surface crossings between higher excited states, which, in turn, will favor intersystem crossings. In consequence, such spin-forbidden crossings will lead to dissociation on triplet excited states, that is, will allow the C–N bond breaking to be the primary decomposition channel. The dissociation curves including the triplet states are shown in Figure S1. Given that the surface crossings observed in this figure are above the excitation energy applied in the experiments, it is likely that reactive crossings occur at geometrical rearrangements where the C–N bond is compressed instead of enlarged as shown in Figure S2 and at geometries where the *C*_2v_ symmetry is broken. This is the case for a related compound (nitrosobenzene) recently studied by us.^62^

Concerning dissociation on the S_0_ state, given that the ground state surface is well separated from the other states, the S_0_ (1^1^A_1_) curve shown in Figure 2 can be considered as a faithful representation of the process leading to the dissociation of nitrobenzene into phenyl and nitrogen dioxide on the ground state. The profile of the S_0_ dissociation corresponds to a typical dissociation of a singlet molecule into two doublet fragments. The enthalpy of dissociation calculated from this calculation (Δ_r_*H*°(0 K) = 307.2 kJ mol^–1^) agrees well with the experimental value^79−81^ (within experimental uncertainty, Table 3). To highlight the importance of the right selection of the active space, we have computed the dissociation surface with two smaller active spaces, that is, CASSCF(16e and 13o) and CASSCF(14e and 11o). While dissociation enthalpy is slightly underestimated by CASSCF(16e and 13o), the same magnitude is hugely overestimated by CASSCF(14e and 11o). More importantly, the profiles obtained with such active spaces, which are shown in Figures S2 and S3, indicate unphysical behavior of the dissociation process; the reason lies in a change in the orbitals that compose the active space. In addition, we have applied the MC-PDFT approximation^42−50^ to calculate the dissociation curves, as shown in Figure 2, where the SCF reference wave function is CASSCF(20e and 17o).

**Table 3:** Enthalpies of Formation in kJ/mol^a^

species	Δ_f_*H*° (0 K)	Δ_f_*H*°(298.15 K)	uncertainty
nitrobenzene (g)	81.4	61.4	±1.4
phenyl (g)	350.35	336.99	±0.53
NO_2_ (g)	36.856	34.049	±0.065

a Data taken from refs (60) and (61).

The obtained results are plotted and shown in Figure S4. In this case, the profiles resulting from these calculations are similar to the MS-CASPT2 ones; however, the dissociation enthalpy is underestimated by ∼18 kJ mol^–1^.

## Conclusions

In this work, the electronic structure of nitrobenzene is studied in order to understand the low-lying singlet and triplet vertical excitation energies and dissociation energies of the molecule on the ground state. This is done at the MS-CASPT2//CASSCF level with a reference active space of 20 electrons distributed in 17 orbitals. The molecule has five singlet valence states in the 4.0–6.0 eV energy range, which correspond to single excited configurations. The calculated vertical excitation energies and dissociation enthalpy agree well with the experimental values in the gas phase [Δ_r_*H*^°^(0 K) = 307.2 kJ mol^–1^]. In addition, it is shown that the reduction of the active space can lead to erroneous results, especially, at the dissociation region because orbital rotations cause a change of the active space along the dissociation reaction coordinate. This fact is a serious drawback and must be taken into account in applying automated procedures for the selection of the active spaces or CASSCF-like methods, which alternately permit the automatic reduction or enlargement of the active spaces.

## Supporting Information Available

Dissociation potential energy curves calculated with reduced active spaces and the MC-PDFT method, vertical excitations calculated with the MC-PDFT approach, and absolute energies of vertical excitations in hartrees (PDF)

## References

[ref1] Galloway D. B.; Bartz J. A.; Huey L. G.; Crim F. F. Pathways and kinetic energy disposal in the photodissociation of nitrobenzene. J. Chem. Phys. 1993, 98, 2107–2114. 10.1063/1.464188.

[ref2] Galloway D. B.; Glenewinkel-Meyer T.; Bartz J. A.; Huey L. G.; Crim F. F. The kinetic and internal energy of NO from the photodissociation of nitrobenzene. J. Chem. Phys. 1994, 100, 1946–1952. 10.1063/1.466547.

[ref3] Gao Z.; Yang M.; Tang C.; Yang F.; Fan X.; Yang R.; Huang Z. Ab initio calculation for isomerization reaction kinetics of nitrobenzene isomers. Chem. Phys. Lett. 2019, 715, 244–251. 10.1016/j.cplett.2018.11.038.

[ref4] Takezaki M.; Hirota N.; Terazima M.; Sato H.; Nakajima T.; Kato S. Nonradiative Relaxation Processes and Electronically Excited States of Nitrobenzene Studied by Picosecond Time-Resolved Transient Grating Method. J. Phys. Chem. A 1997, 101, 3443–3448. 10.1021/jp963095t.

[ref5] Vidal B.; Murrell J. N. Effect of Solvent on position of First Absorption-Band of Nitrobenzene. Chem. Phys. Lett. 1975, 31, 46–47. 10.1016/0009-2614(75)80054-6.

[ref6] Lin M.-F.; Lee Y. T.; Ni C.-K.; Xu S.; Lin M. C. Photodissociation Dynamics of Nitrobenzene and o-Nitrotoluene. J. Chem. Phys. 2007, 126, 064310 10.1063/1.2435351. 17313218

[ref7] Nagakura S.; Kojima M.; Maruyama Y. Electronic spectra and electronic structures of nitrobenzene and nitromesitylene. J. Mol. Spectrosc. 1964, 13, 174–192. 10.1016/0022-2852(64)90066-9.

[ref8] Marshall A.; Clark A.; Jennings R.; Ledingham K. W. D.; Singhal R. P. Wavelength-Dependent Laser-Induced Fragmentation of Nitrobenzene. Int. J. Mass Spectrom. Ion Processes 1992, 112, 273–283. 10.1016/0168-1176(92)80009-p.

[ref9] Sinha H. K.; Yates K. Ground-state and excited-state dipole-moments of some nitroaromatics - evidence for extensive charge-transfer in twisted nitrobenzene systems. J. Chem. Phys. 1990, 93, 7085–7093. 10.1063/1.459431.

[ref10] Kröhl O.; Malsch K.; Swiderek P. The electronic states of nitrobenzene: electron-energy-loss spectroscopy and CASPT2 calculations. Phys. Chem. Chem. Phys. 2000, 2, 947–953. 10.1039/a909290k.

[ref11] Blackshaw K. J.; Ortega B. I.; Quartey N.-K.; Fritzeen W. E.; Korb R. T.; Ajmani A. K.; Montgomery L.; Marracci M.; Vanegas G. G.; Galvan J.; Sarvas Z.; Petit A. S.; Kidwell N. M. Nonstatistical Dissociation Dynamics of Nitroaromatic Chromophores. J. Phys. Chem. A 2019, 123, 4262–4273. 10.1021/acs.jpca.9b02312. 31038954

[ref12] Mewes J.-M.; Jovanović V.; Marian C. M.; Dreuw A. On the molecular mechanism of non-radiative decay of nitrobenzene and the unforeseen challenges this simple molecule holds for electronic structure theory. Phys. Chem. Chem. Phys. 2014, 16, 12393–12406. 10.1039/c4cp01232a. 24827580

[ref13] Giussani A.; Worth G. A. How important is roaming in the photodegradation of nitrobenzene?. Phys. Chem. Chem. Phys. 2020, 22, 15945–15952. 10.1039/d0cp02077j. 32572418

[ref14] Giussani A.; Worth G. A. Similar chemical structures, dissimilar triplet quantum yields: a CASPT2 model rationalizing the trend of triplet quantum yields in nitroaromatic systems. Phys. Chem. Chem. Phys. 2019, 21, 10514–10522. 10.1039/c9cp00705a. 31070625

[ref15] Giussani A.; Worth G. A. Insights into the Complex Photophysics and Photochemistry of the Simplest Nitroaromatic Compound: A CASPT2//CASSCF Study on Nitrobenzene. J. Chem. Theory Comput. 2017, 13, 2777–2788. 10.1021/acs.jctc.6b01149. 28437102

[ref16] Takezaki M.; Hirota N.; Terazima M.; Sato H.; Nakajima T.; Kato S. Geometries and Energies of Nitrobenzene Studied by CAS-SCF Calculations. J. Phys. Chem. A 1997, 101, 5190–5195. 10.1021/jp970937v.

[ref17] Zobel J. P.; Nogueira J. J.; González L. Quenching of Charge Transfer in Nitrobenzene Induced by Vibrational Motion. J. Phys. Chem. Lett. 2015, 6, 3006–3011. 10.1021/acs.jpclett.5b00990. 26267195

[ref18] Kreplin D. A.; Knowles P. J.; Werner H.-J. Second-order MCSCF optimization revisited. I. Improved algorithms for fast and robust second-order CASSCF convergence. J. Chem. Phys. 2019, 150, 194106 10.1063/1.5094644. 31117783

[ref19] Kreplin D. A.; Knowles P. J.; Werner H.-J. MCSCF optimization revisited. II. Combined first- and second-order orbital optimization for large molecules. J. Chem. Phys. 2020, 152, 074102 10.1063/1.5142241. 32087666

[ref20] Levine D. S.; Hait D.; Tubman N. M.; Lehtola S.; Whaley K. B.; Head-Gordon M. CASSCF with Extremely Large Active Spaces Using the Adaptive Sampling Configuration Interaction Method. J. Chem. Theory Comput. 2020, 16, 2340–2354. 10.1021/acs.jctc.9b01255. 32109055

[ref21] Helmich-Paris B. CASSCF linear response calculations for large open-shell molecules. J. Chem. Phys. 2019, 150, 174121 10.1063/1.5092613. 31067879

[ref22] Jeong W.; Stoneburner S. J.; King D.; Li R.; Walker A.; Lindh R.; Gagliardi L. Automation of Active Space Selection for Multireference Methods via Machine Learning on Chemical Bond Dissociation. J. Chem. Theory Comput. 2020, 16, 2389–2399. 10.1021/acs.jctc.9b01297. 32119542

[ref23] Vogiatzis K. D.; Ma D.; Olsen J.; Gagliardi L.; de Jong W. A. Pushing configuration-interaction to the limit: Towards massively parallel MCSCF calculations. J. Chem. Phys. 2017, 147, 184111 10.1063/1.4989858. 29141437

[ref24] Tóth Z.; Pulay P. Comparison of Methods for Active Orbital Selection in Multiconfigurational Calculations. J. Chem. Theory Comput. 2020, 16, 7328–7341. 10.1021/acs.jctc.0c00123. 33170653 PMC7726099

[ref25] King D. S.; Gagliardi L. A Ranked-Orbital Approach to Select Active Spaces for High- Throughput Multireference Computation. J. Chem. Theory Comput. 2021, 17, 2817–2831. 10.1021/acs.jctc.1c00037. 33860669

[ref26] Blahous C. P. III; Yates B. F.; Xie Y.; Schaefer H. F. III Symmetry breaking in the NO_2_ σ radical: Construction of the ^2^A_1_ and ^2^B_2_ states with C_s_ symmetry complete active space self-consistent-field wave functions. J. Chem. Phys. 1990, 93, 8105–8109. 10.1063/1.459712.

[ref27] Arenas J. F.; Otero J. C.; Peláez D.; Soto J. Role of surface crossings in the photochemistry of nitromethane. J. Chem. Phys. 2005, 122, 084324 10.1063/1.1851977. 15836056

[ref28] Arenas J. F.; Otero J. C.; Peláez D.; Soto J.; Serrano-Andrés L. Multiconfigurational second-order perturbation study of the decomposition of the radical anion of nitromethane. J. Chem. Phys. 2004, 121, 4127–4132. 10.1063/1.1772357. 15332959

[ref29] Soto J.; Peláez D.; Otero J. C.; Avila F. J.; Arenas J. F. Photodissociation mechanism of methyl nitrate. A study with the multistate second-order multiconfigurational perturbation theory. Phys. Chem. Chem. Phys. 2009, 11, 2631–2639. 10.1039/b820646e. 19421519

[ref30] Arenas J. F.; Otero J. C.; Peláez D.; Soto J. Photodissociation mechanism of nitramide: A CAS-SCF and MS-CASPT2 study. J. Phys. Chem. A 2005, 109, 7172–7180. 10.1021/jp058100k. 16834081

[ref31] Ruano C.; Otero J. C.; Arenas J. F.; Soto J. Multiconfigurational second-order perturbation study of the photochemical decomposition of methyl thionitrite. Chem. Phys. Lett. 2012, 553, 17–20. 10.1016/j.cplett.2012.09.060.

[ref32] Ndengué S.; Quintas-Sánchez E.; Dawes R.; Osborn D. The Low-Lying Electronic States of NO2: Potential Energy and Dipole Surfaces, Bound States, and Electronic Absorption Spectrum. J. Phys. Chem. A 2021, 125, 5519–5533. 10.1021/acs.jpca.1c03482. 34114826

[ref33] Roos B. O. Advances in Chemical Physics; Ab initio Methods in Quantum Chemistry II; Lawley K. P., Ed.; John Wiley & Sons: Chichester, U.K., 1987; Chapter 69; p 399.

[ref34] Roos B. O.; Taylor P. R.; Sigbahn P. E. M. A complete active space SCF method (CASSCF) using a density-matrix formulated super-CI approach. Chem. Phys. 1980, 48, 157–173. 10.1016/0301-0104(80)80045-0.

[ref35] Roos B. O. The complete active space SCF method in a Fock-matrix-based super-CI formulation. Int. J. Quantum Chem. 1980, 18, 175–189. 10.1002/qua.560180822.

[ref36] Siegbahn P. E. M.; Almlöf J.; Heiberg A.; Roos B. O. The complete active space SCF (CASSCF) method in a Newton-Raphson formulation with application to the HNO molecule. J. Chem. Phys. 1981, 74, 2384–2396. 10.1063/1.441359.

[ref37] Werner H. J.; Meyer W. A quadratically convergent multiconfiguration-self-consistent field method with simultaneous optimization of orbitals and CI coefficients. J. Chem. Phys. 1980, 73, 2342–2356. 10.1063/1.440384.

[ref38] Werner H. J.; Meyer W. A quadratically convergent MCSCF method for the simultaneous optimization of several states. J. Chem. Phys. 1981, 74, 5794–5801. 10.1063/1.440892.

[ref39] Olsen J. The CASSCF Method: A Perspective and Commentary. Int. J. Quantum Chem. 2011, 111, 3267–3272. 10.1002/qua.23107.

[ref40] Roos B. O.; Andersson K.; Fülscher M. P.; Malmqvist P. Å.; Serrano-Andrés L.; Pierloot K.; Merchán M. Multiconfigurational perturbation theory: Applications in electronic spectroscopy. Adv. Chem. Phys. 1996, 93, 219–331. 10.1002/9780470141526.ch5.

[ref41] Finley J.; Malmqvist P.-Å.; Roos B. O.; Serrano-Andrés L. The multi-state CASPT2 method. Chem. Phys. Lett. 1998, 288, 299–306. 10.1016/s0009-2614(98)00252-8.

[ref42] Stoneburner S. J.; Truhlar D. G.; Gagliardi L. Transition Metal Spin-State Energetics by MC-PDFT with High Local Exchange. J. Phys. Chem. A 2020, 124, 1187–1195. 10.1021/acs.jpca.9b10772. 31962045

[ref43] Li Manni G.; Carlson R. K.; Luo S.; Ma D.; Olsen J.; Truhlar D. G.; Gagliardi L. Multiconfiguration Pair-Density Functional Theory. J. Chem. Theory Comput. 2014, 10, 3669–3680. 10.1021/ct500483t. 26588512

[ref44] Gagliardi L.; Truhlar D. G.; Li Manni G.; Carlson R. K.; Hoyer C. E.; Bao J. L. Multiconfiguration Pair-Density Functional Theory: A New Way to Treat Strongly Correlated Systems. Acc. Chem. Res. 2017, 50, 66–73. 10.1021/acs.accounts.6b00471. 28001359

[ref45] Carlson R. K.; Li Manni G.; Sonnenberger A. L.; Truhlar D. G.; Gagliardi L. Multiconfiguration Pair-Density Functional Theory: Barrier Heights and Main Group and Transition Metal Energetics. J. Chem. Theory Comput. 2015, 11, 82–90. 10.1021/ct5008235. 26574206

[ref46] Sand A. M.; Kidder K. M.; Truhlar D. G.; Gagliardi L. Calculation of Chemical Reaction Barrier Heights by Multiconfiguration Pair-Density Functional Theory with Correlated Participating Orbitals. J. Phys. Chem. A 2019, 123, 9809–9817. 10.1021/acs.jpca.9b08134. 31609619

[ref47] Li S. J.; Gagliardi L.; Truhlar D. G. Extended separated-pair approximation for transition metal potential energy curves. J. Chem. Phys. 2020, 152, 124118 10.1063/5.0003048. 32241117

[ref48] Dong S. S.; Gagliardi L.; Truhlar D. G. Nature of the 1(1)B(u) and 2(1)A(g) Excited States of Butadiene and the Goldilocks Principle of Basis Set Diffuseness. J. Chem. Theory Comput. 2019, 15, 4591–4601. 10.1021/acs.jctc.9b00549. 31306007

[ref49] Zhou C.; Gagliardi L.; Truhlar D. G. State-interaction pair density functional theory for locally avoided crossings of potential energy surfaces in methylamine. Phys. Chem. Chem. Phys. 2019, 21, 13486–13493. 10.1039/c9cp02240f. 31204766

[ref50] Sharma P.; Truhlar D. G.; Gagliardi L. Active Space Dependence in Multiconfiguration Pair-Density Functional Theory. J. Chem. Theory Comput. 2018, 14, 660–669. 10.1021/acs.jctc.7b01052. 29301088

[ref51] MOLCAS 8.4 Veryazov V.; Widmark P.-O.; Serrano-Andrés L.; Lindh R.; Roos B. O. 2MOLCAS as a development platform for quantum chemistry software. Int. J. Quantum Chem. 2004, 100, 626–635. 10.1002/qua.20166.

[ref52] Aquilante F.; Autschbach J.; Carlson R. K.; Chibotaru L. F.; Delcey M. G.; De Vico L.; Fdez. Galván I.; Ferré N.; Frutos L. M.; Gagliardi L.; Garavelli M.; Giussani A.; Hoyer C. E.; Li Manni G.; Lischka H.; Ma D.; Malmqvist P. Å.; Müller T.; Nenov A.; Olivucci M.; Pedersen T. B.; Peng D.; Plasser F.; Pritchard B.; Reiher M.; Rivalta I.; Schapiro I.; Segarra-Martí J.; Stenrup M.; Truhlar D. G.; Ungur L.; Valentini A.; Valentini S.; Veryazov V.; Vysotskiy V. P.; Weingart O.; Zapata F.; Lindh R.; Lindh R. Molcas 8: New capabilities for multiconfigurational quantum chemical calculations across the periodic table. J. Comput. Chem. 2016, 37, 506–541. 10.1002/jcc.24221. 26561362

[ref53] Roos B. O.; Lindh R.; Malmqvist P.-Å.; Veryazov V.; Widmark P.-O. Main group atoms and dimers studied with a new relativistic ANO basis set. J. Phys. Chem. A 2004, 108, 2851–2858. 10.1021/jp031064+.

[ref54] Roos B. O.; Lindh R.; Malmqvist P.-Å.; Veryazov V.; Widmark P.-O. New relativistic ANO basis sets for transition metal atoms. J. Phys. Chem. A 2005, 109, 6575–6579. 10.1021/jp0581126. 16834004

[ref55] Zobel J. P.; Widmark P.-O.; Veryazov V. The ANO-R Basis Set. J. Chem. Theory Comput. 2020, 16, 278–294. 10.1021/acs.jctc.9b00873. 31738554

[ref56] Peláez D.; Arenas J. F.; Otero J. C.; Soto J. A complete active space self-consistent field study of the photochemistry of nitrosamine. J. Chem. Phys. 2006, 125, 164311 10.1063/1.2360259. 17092077

[ref57] Algarra M.; Soto J. Insights into the Thermal and Photochemical Reaction Mechanisms of Azidoacetonitrile. Spectroscopic and MS-CASPT2 Calculations. ChemPhysChem 2020, 21, 1126–1133. 10.1002/cphc.202000201. 32289197

[ref58] Louleb M.; Latrous L.; Ríos Á.; Zougagh M.; Rodríguez-Castellón E.; Algarra M.; Soto J. Detection of Dopamine in Human Fluids Using N-Doped Carbon Dots. ACS Appl. Nano Mater. 2020, 3, 8004–8011. 10.1021/acsanm.0c01461.

[ref59] Algarra M.; Moreno V.; Lázaro-Martínez J. M.; Rodríguez-Castellón E.; Soto J.; Morales J.; Benítez A. Insights into the formation of N doped 3D-graphene quantum dots. Spectroscopic and computational approach. J. Colloid Interface Sci. 2020, 561, 678–686. 10.1016/j.jcis.2019.11.044. 31761465

[ref60] Kopec S.; Martinez-Nunez E.; Soto J.; Pelaez D. vdW-TSSCDS-An automated and global procedure for the computation of stationary points on intermolecular potential energy surfaces. Int. J. Quantum Chem. 2019, 119, e26008 10.1002/qua.26008.

[ref61] Algarra M.; Soto J.; Pinto da Silva L.; Pino-González M. S.; Rodríguez-Borges J. E.; Mascetti J.; Borget F.; Reisi-Vanani A.; Luque R. Insights into the Photodecomposition of Azidomethyl Methyl Sulfide: A S-2/S-1 Conical Intersection on Nitrene Potential Energy Surfaces Leading to the Formation of S-Methyl-N-sulfenylmethanimine. J. Phys. Chem. A 2020, 124, 1911–1921. 10.1021/acs.jpca.9b11157. 32053376

[ref62] Soto J.; Peláez D.; Otero J. C. A SA-CASSCF and MS-CASPT2 study on the electronic structure of nitrosobenzene and its relation to its dissociation dynamics. J. Chem. Phys. 2021, 154, 044307 10.1063/5.0033181. 33514099

[ref63] Soto J.; Otero J. C. Conservation of El-Sayed’s Rules in the Photolysis of Phenyl Azide: Two Independent Decomposition Doorways for Alternate Direct Formation of Triplet and Singlet Phenylnitrene. J. Phys. Chem. A 2019, 123, 9053–9060. 10.1021/acs.jpca.9b06915. 31573200

[ref64] Aranda D.; Avila F. J.; López-Tocón I.; Arenas J. F.; Otero J. C.; Soto J. An MS-CASPT2 study of the photodecomposition of 4-methoxyphenyl azide: role of internal conversion and intersystem crossing. Phys. Chem. Chem. Phys. 2018, 20, 7764–7771. 10.1039/c8cp00147b. 29504003

[ref65] Soto J.; Otero J. C.; Avila F. J.; Peláez D. Conical intersections and intersystem crossings explain product formation in photochemical reactions of aryl azides. Phys. Chem. Chem. Phys. 2019, 21, 2389–2396. 10.1039/c8cp06974c. 30649110

[ref66] Allouche A. R. Gabedit-A Graphical User Interface for Computational Chemistry Softwares. J. Comput. Chem. 2011, 32, 174–182. 10.1002/jcc.21600. 20607691

[ref67] Schaftenaar G.; Noordik J. H. Molden: a pre- and post-processing program for molecular and electronic structures. J. Comput.-Aided Mol. Des. 2000, 14, 123–134. 10.1023/a:1008193805436. 10721501

[ref68] Bode B. M.; Gordon M. S. MacMolPlt: A graphical user interface for GAMESS. J. Mol. Graphics Modell. 1998, 16, 133–138. 10.1016/s1093-3263(99)00002-9. 10434252

[ref69] Gagliardi L.; Lindh R.; Karlström G. Local properties of quantum chemical systems: The LoProp approach. J. Chem. Phys. 2004, 121, 4494–4500. 10.1063/1.1778131. 15332879

[ref70] Soto J.; Avila F. J.; Otero J. C.; Arenas J. F. Comment on “Multiconfigurational perturbation theory can predict a false ground state by C. Camacho, R. Cimiraglia and H. A. Witek”. Phys. Chem. Chem. Phys. 2011, 13, 7230–7231. 10.1039/C0CP01917H. 21394350

[ref71] Bernardi F.; Olivucci M.; Robb M. A.; Vreven T.; Soto J. An ab initio study of the photochemical decomposition of 3,3-dimethyldiazirine. J. Org. Chem. 2000, 65, 7847–7857. 10.1021/jo000856m. 11073590

[ref72] Arenas J. F.; López-Tocón I.; Otero J. C.; Soto J. Carbene formation in its lower singlet state from photoexcited 3H-diazirine or diazomethane. A combined CASPT2 and ab initio direct dynamics trajectory study. J. Am. Chem. Soc. 2002, 124, 1728–1735. 10.1021/ja010750o. 11853450

[ref73] Domenicano A.; Schultz G. r.; Hargittai I. n.; Colapietro M.; Portalone G.; George P.; Bock C. W. Molecular Structure of Nitrobenzene in the Planar and Orthogonal Conformations A Concerted Study by Electron Diffraction, X-Ray Crystallography, and Molecular Orbital Calculations. Struct. Chem. 1990, 1, 107–122. 10.1007/bf00675790.

[ref74] Miller W. H.; Handy N. C.; Adams J. E. Reaction-path hamiltonian for polyatomic-molecules. J. Chem. Phys. 1980, 72, 99–112. 10.1063/1.438959.

[ref75] Bearpark M.; Robb M.; Yamamoto N. A CASSCF study of the cyclopentadienyl radical: conical intersections and the Jahn-Teller effect. Spectrochim. Acta, Part A 1999, 55, 639–646. 10.1016/s1386-1425(98)00267-4.

[ref76] Arenas J. F.; Marcos J. I.; López-Tocón I.; Otero J. C.; Soto J. Potential-energy surfaces related to the thermal decomposition of ethyl azide: The role of intersystem crossings. J. Chem. Phys. 2000, 113, 2282–2289. 10.1063/1.482043.

[ref77] Weisskopf V.; Wigner E. Calculation of the natural width of line based on the Diracsch’s theory of light. Z. Physik. 1930, 63, 54–73. 10.1007/bf01336768.

[ref78] Colson S. D.; Bernstein E. R. First and Second Triplets of Solid Benzene. J. Chem. Phys. 1965, 43, 2661–2669. 10.1063/1.1697192.

[ref79] Ruscic B.; Bross D. H. Active Thermochemical Tables (ATcT) values based on ver. 1.122p of the Thermochemical Network; available at https://atct.anl.gov/Thermochemical%20Data/version%201.110 (2020).

[ref80] Ruscic B.; Pinzon R. E.; Morton M. L.; von Laszevski G.; Bittner S. J.; Nijsure S. G.; Amin K. A.; Minkoff M.; Wagner A. F. Introduction to active thermochemical tables: Several “key” enthalpies of formation revisited. J. Phys. Chem. A 2004, 108, 9979–9997. 10.1021/jp047912y.

[ref81] Ruscic B.; Pinzon R. E.; Laszewski G. v.; Kodeboyina D.; Burcat A.; Leahy D.; Montoy D.; Wagner A. F. Active Thermochemical Tables: thermochemistry for the 21st century. J. Phys.: Conf. Ser. 2005, 16, 561–570. 10.1088/1742-6596/16/1/078.

