# Supplementary material for: Electronic Structure of Nitrobenzene: A Benchmark Example of the Accuracy of the Multi-State CASPT2 Theory
Source: J Phys Chem A. 2021 Oct 22;125(43):9431–7. doi: 10.1021/acs.jpca.1c04595 (PMC8573753; doi:10.1021/acs.jpca.1c04595)
Supplement: Supplementary file 1 — jp1c04595_si_001.pdf [file jp1c04595_si_001.pdf]

Supporting Information

Electronic Structure of Nitrobenzene: A Benchmark Example of the Accuracy of the Multi-State CASPT2 Theory

by

Juan Soto*,[a] and Manuel Algarra,[b]

[a]Department of Physical Chemistry, Faculty of Science, University of Málaga, 29071, Málaga, Spain

[b]Department of Inorganic Chemistry, Faculty of Science, University of Málaga, 29071, Málaga, Spain

E-mail: soto@uma.es

CONTENTS

Table S1 Vertical excitation energies in eV of the singlet states of nitrobenzene (C_{2v}, MS-CASPT2) at the CASSCF(20e, 17o)/ANO-RCC optimized geometry. S3

Table S2 Experimental and calculated geometrical parameters of nitrobenzene...........S4

Table S3 Vertical excitation energies in eV of the low-lying singlet states of nitrobenzene calculated with MS-CASPT2 and MC-PDFT methods.. ....S5

Table S4 Absolute energies in hartrees of singlet and triplet states included in Table 1... S6

Figure S1 SA3-CASSCF/ANO-RCC potential energy curves of the low-lying singlet and triplet states of nitrobenzene leading to dissociation into phenyl radical and nitrogen dioxide...S7

Figure S2 MS-CASPT2/ANO-RCC potential energy curves of the low-lying A_1 singlet states of nitrobenzene leading to dissociation into phenyl radical and nitrogen dioxide. In red, SA3-CASSCF(16e, 13o) reference wave function; in blue, SA3-CASSCF(20e, 17o) reference wave function...S8

Figure S3 (a) ANO-RCC interpolations lines of the potential energy surface of phenyl azide leading from M1 to ($3^1A'/2^3A''$) ISC0 (Blue: Singlet; Red: Triplet. Solid [MS-CASPT2], dotted [CASPT2], broken line [CASSCF])] (b) Spin-orbit coupling constant along the interpolation line S9

Figure S4 MC-PDFT/ftrevPBE/ANO-RCC potential energy curves of the low-lying singlet states of nitrobenzene leading to dissociation into phenyl radical and nitrogen dioxide. Reference wave function: SA3-CASSCF(20e, 17o). A_1 states (blue lines); A_2 states (orange dotted lines); B_1 states (green lines); B_2 states (orange solid lines). S10

Table S1. Vertical excitation energies in eV of the singlet states of nitrobenzene (C_{2v}, MS-CASPT2) at the CASSCF(20e, 17o)/ANO-RCC optimized geometry.[a,b]

Transition	ΔE	*OS*[c]	*Configuration*[d]	*Weight*[e]
$1A_1 \rightarrow 2A_1$	**5.22**	2.86-01	$[\pi_3(bz)]^1[\pi^*(NO_2)]^1$	72
$1A_1 \rightarrow 3A_1$	7.72	1.04-02	$[\pi_1(bz)]^1[\pi^*(NO_2)]^1$	33
			$[\pi_3(bz)]^0[\pi^*(NO_2)]^2$	15
$1A_1 \rightarrow 1A_2$	3.94	<1.0-05	$[n\sigma]^1[\pi^*(NO_2)]^1$	69
$1A_1 \rightarrow 2A_2$	7.12	3.85-03	$[n\sigma]^1[\pi_3(bz)]^1[\pi^*(NO_2)]^2$	24
			$[n\sigma]^1[\pi_3^*(bz)]^1$	34
$1A_1 \rightarrow 3A_2$	7.41	4.02-05	$[\sigma_1(NO_2)]^1[\sigma_2^*(NO_2)]^1$	57
$1A_1 \rightarrow 1B_2$	**4.37**	1.42-04	$[\sigma_1(NO_2)]^1[\pi^*(NO_2)]^1$	66
$1A_1 \rightarrow 2B_2$	7.14	9.07-04	$[n\sigma]^1[\pi_2^*(bz)]^1$	51
			$[n\sigma]^1[\pi_2(bz)]^1[\pi^*(NO_2)]^2$	15
$1A_1 \rightarrow 3B_2$	7.57	4.59-03	$[\sigma_1(NO_2)]^1[\pi_3(bz)]^1[\pi^*(NO_2)]^2$	18
			$[\sigma_1(NO_2)]^1[\pi_3^*(bz)]^1$	40
$1A_1 \rightarrow 1B_1$	**4.79**	4.59-03	$[\pi_3(bz)]^1[\pi_2^*(bz)]^1$	21
			$[\pi_2(bz)]^1[\pi^*(NO_2)]^1$	43
$1A_1 \rightarrow 2B_1$	**5.98**	3.88-02	$[n\pi]^1[\pi^*(NO_2)]^1$	47
			$[n\pi]^1[\pi_3(bz)]^1[\pi^*(NO_2)]^2$	
$1A_1 \rightarrow 3B_1$	7.12	8.29-02	$[\pi_3(bz)]^1[\pi_2^*(bz)]^1$	17

[a]CASSCF(20e, 17o)/ANO-RCC optimized geometry. [b]SA3-CASSCF(20e, 17o)/ANO-RCC references wave function. IPEA=0.25. Imaginary shift = 0.1. [c]Oscillator strength. [d]MS-CASPT2 main electronic configurations of the excited states referred to the ground state configuration. [e]Weight of the configuration in %. Only contributions greater than 15% are included.

Table S2. Experimental and calculated geometrical parameters of nitrobenzene.

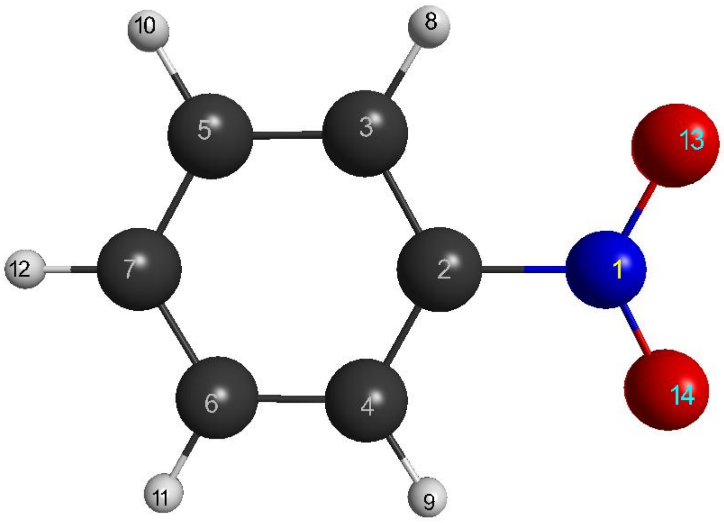

Internal Coordinate	Experimental[a]	MP2[b]	CASSCF[b]
R[2,1]	1.486	1.472	1.504
R[3,2]	1.399	1.388	1.386
R[5,3]	1.399	1.392	1.390
R[7,5]	1.399	1.394	1.392
R[8,3]	1.093	1.079	1.069
R[10,5]	1.093	1.081	1.072
R[12,7]	1.093	1.081	1.072
R[13,1]	1.223	1.228	1.223
A[4,2,3]	123.4	122.7	122.4
A[5,3,2]	117.6	118.2	118.5
A[7,5,3]	120.6	120.4	120.3
A[8,3,2]	-	119.7	120.2
A[10,5,3]	-	119.5	119.6
A[12,7,5]	119.9	120.0	119.9
A[13,1,2]	117.3	117.4	117.4

[a]Reference: [Domenicano, A.; Schutz, G.; Hargittai, I.; Colapietro, M.; Por-talone, G.; George, P.; Bock, C. W. Molecular Structure of Ni-trobenzene in the Planar and Orthogonal Conformations A Concerted Study by Electron Diffraction, X-Ray Crystallography Calculations. *Struct. Chem.* **1990**, *1*, 107-122.]. [b]MP2/def2-TZVPP. [c]CASSCF(20**e**, 17**o**)/ANO-RCC. Theoretical parameters: C_{2v} symmetry. R: internuclear distances in Å. A: bond angles in degrees.

Table S3. Vertical excitation energies in eV of the low-lying singlet states of nitrobenzene calculated with MS-CASPT2 and MC-PDFT methods.[a,b]

Transition	MS(1)[c]	MS(2)[d]	MC(3)[e]	MC(4)[f]	MC(5)[g]	MC(6)[h]	MC(7)[i]	Exp.
$1A_1 \rightarrow 2A_1$	5.11	4.75	4.76	4.80	4.76	4.82	4.69	5.00
$1A_1 \rightarrow 3A_1$	7.60	7.16	7.35	7.30	7.37	7.29	7.29	
$1A_1 \rightarrow 1A_2$	3.83	3.62	3.90	3.93	3.90	3.93	3.90	3.54
$1A_1 \rightarrow 2A_2$	7.00	6.61	7.19	7.20	7.20	7.19	7.18	
$1A_1 \rightarrow 3A_2$	7.36	6.95	7.36	7.42	7.37	7.42	7.44	
$1A_1 \rightarrow 1B_2$	4.30	4.07	4.33	4.36	4.33	4.36	4.34	
$1A_1 \rightarrow 2B_2$	7.02	6.57	7.09	7.13	7.11	7.13	7.14	
$1A_1 \rightarrow 3B_2$	7.51	7.11	7.52	7.56	7.54	7.56	7.54	
$1A_1 \rightarrow 1B_1$	4.72	4.34	4.87	4.78	4.88	4.76	4.77	4.43
$1A_1 \rightarrow 2B_1$	5.81	5.60	5.82	5.80	5.84	5.80	5.77	
$1A_1 \rightarrow 3B_1$	7.04	6.50	6.77	6.72	6.79	6.71	6.69	6.42

[a]MP2/def2-TZVPP optimized geometry. [b]SA3-CASSCF(20**e**, 17**o**)/ANO-RCC reference wave function. [c]MS-CASPT2 IPEA=0.25. [d]MS-CASPT2 IPEA=0.0 Imaginary shift = 0.1. [e]MC-PDFT/tPBE. [f]MC-PDFT/ftPBE. [g]MC-PDFT/trevPBE. [h]MC-PDFT/ftrevPBE. [i]MC-PDFT/ftBLYP.

Table S4. Absolute energies in hartrees of singlet and triplet states included in Table 1.

State	CASSCF	MS-CASPT2
1^1A_1	-434.82700604	-436.19345665
2^1A_1	-434.58439053	-436.00551384
3^1A_1	-434.54459844	-435.91422472
1^1A_2	-434.67399405	-436.05268702
2^1A_2	-434.56141357	-435.93638098
3^1A_2	-434.53090222	-435.92298712
1^1B_2	-434.65410806	-436.03527854
2^1B_2	-434.54577612	-435.93550540
3^1B_2	-434.53872490	-435.91746951
1^1B_1	-434.65194077	-436.01982817
2^1B_1	-434.59644245	-435.98006312
3^1B_1	-434.54619364	-435.93477933
1^3A_1	-434.69576329	-436.04524898
2^3A_1	-434.65459264	-436.01902428
3^3A_1	-434.58219672	-435.94046700
1^3A_2	-434.68342772	-436.06008099
2^3A_2	-434.56287899	-435.93467186
3^3A_2	-434.52916833	-435.89627993
1^3B_2	-434.65819809	-436.03989842
2^3B_2	-434.54629001	-435.93440860
3^3B_2	-434.54236839	-435.91891814
1^3B_1	-434.68500759	-436.06362144
2^3B_1	-434.65251479	-436.02554678
3^3B_1	-434.58349516	-435.97197678

[a]MP2/def2-TZVPP optimized geometry. [b]SA3-CASSCF reference wave function, IPEA=0.25. Imaginary shift = 0.1.

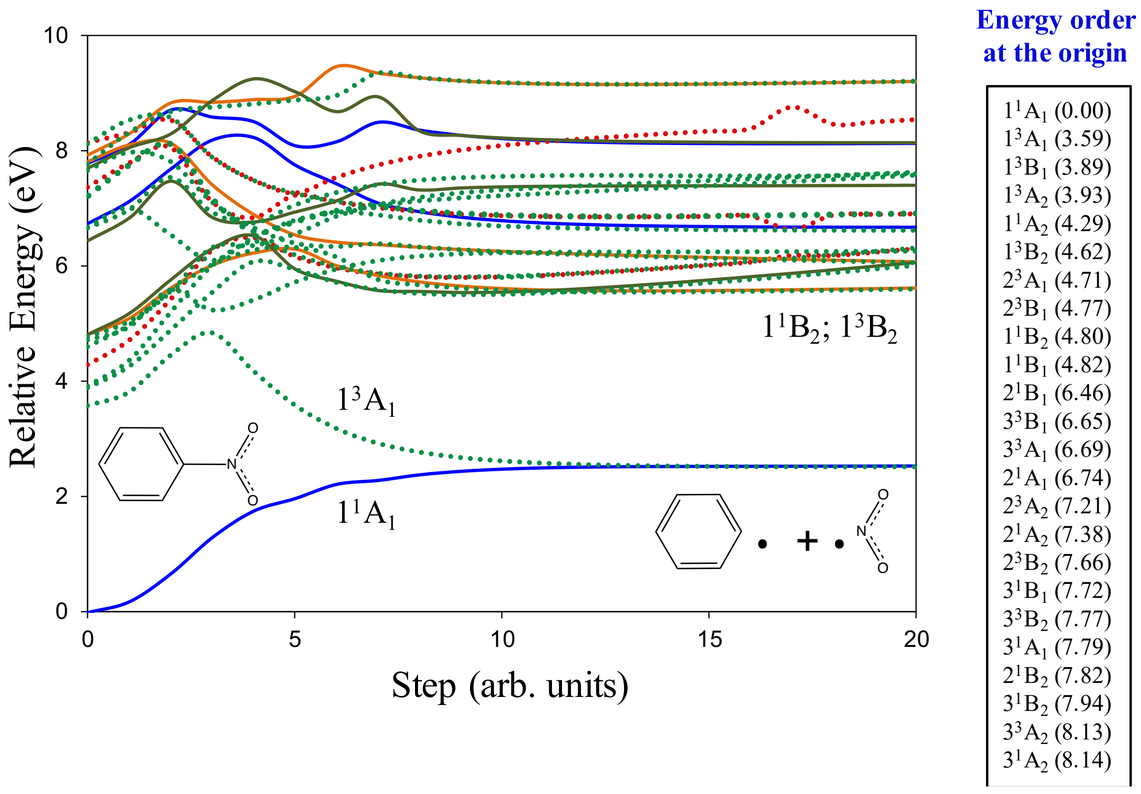

Figure S1. SA3-CASSCF/ANO-RCC potential energy curves of the low-lying singlet and triplet states of nitrobenzene leading to dissociation into phenyl radical and nitrogen dioxide. Singlet: A_1 states (blue lines); A_2 states (orange dotted lines); B_1 states (green lines); B_2 states (orange solid lines). Triplet: green dotted lines.

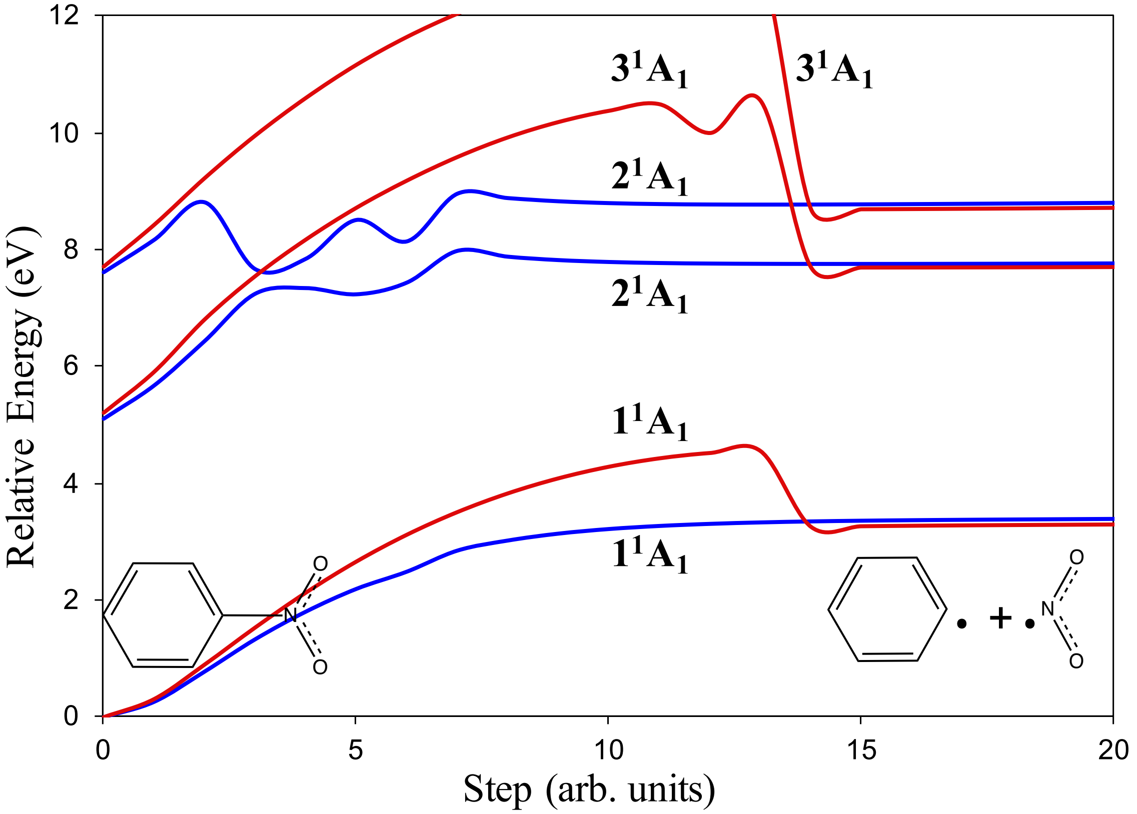

Figure S2. MS-CASPT2/ANO-RCC potential energy curves of the low-lying A_1 singlet states of nitrobenzene leading to dissociation into phenyl radical and nitrogen dioxide. In red, SA3-CASSCF(16e, 13o) reference wave function; in blue, SA3-CASSCF(20e, 17o) reference wave function.

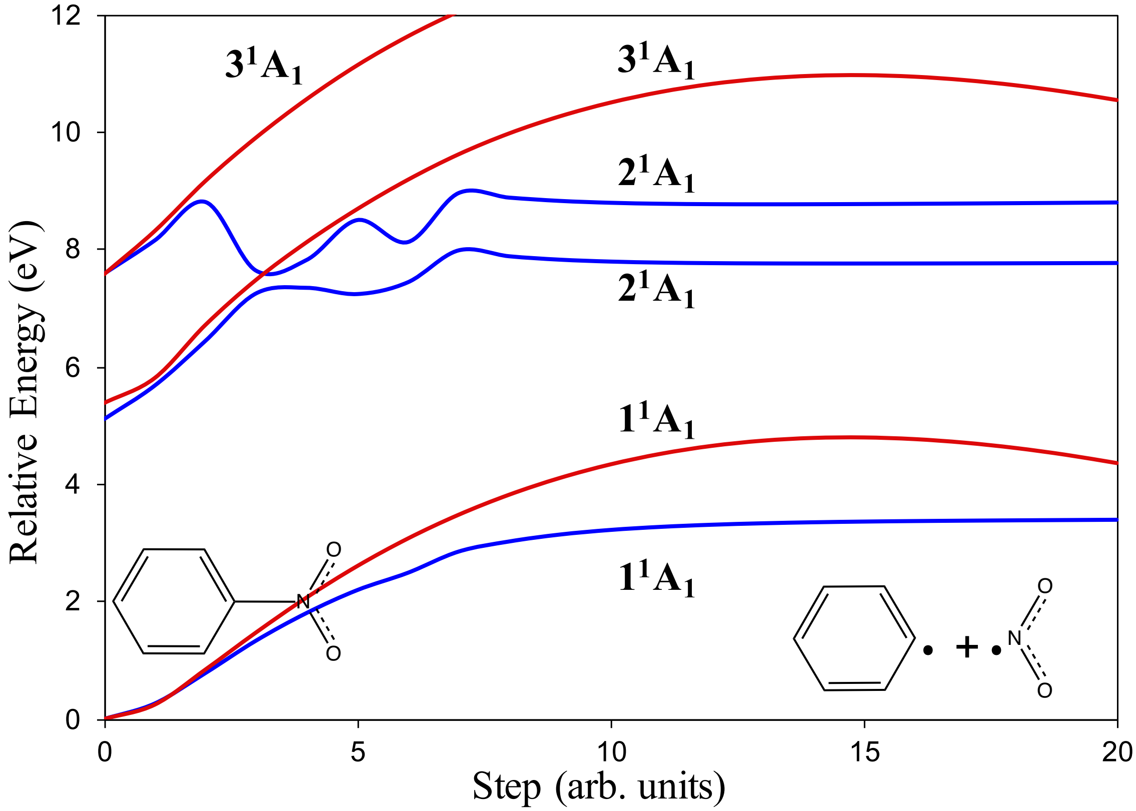

Figure S3. MS-CASPT2/ANO-RCC potential energy curves of the low-lying A_1 singlet states of nitrobenzene leading to dissociation into phenyl radical and nitrogen dioxide. In red, SA3-CASSCF(14e, 11o) reference wave function; in blue, SA3-CASSCF(20e, 17o) reference wave function.

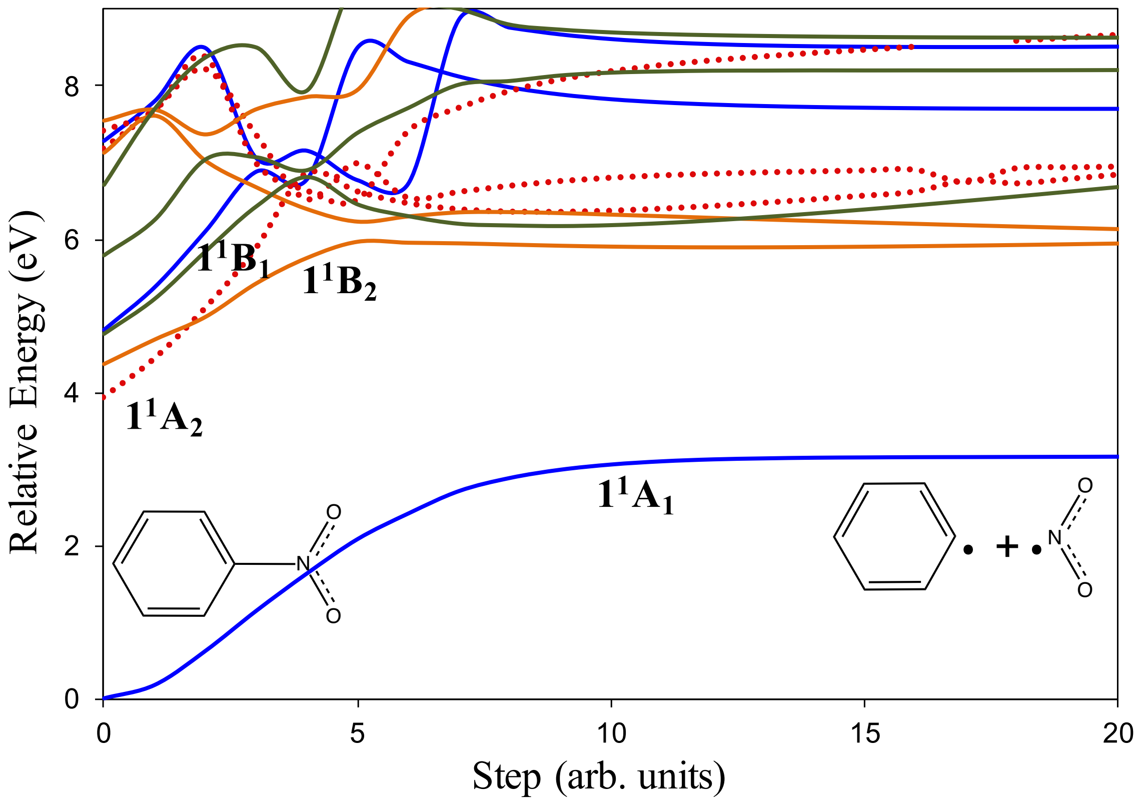

Figure S4. MC-PDFT/ftrevPBE/ANO-RCC potential energy curves of the low-lying singlet states of nitrobenzene leading to dissociation into phenyl radical and nitrogen dioxide. Reference wave function: SA3-CASSCF(20e, 17o). A_1 states (blue lines); A_2 states (orange dotted lines); B_1 states (green lines); B_2 states (orange solid lines).

Cartesian coordinates of nitrobenzene in Å. CASSCF(20e,17o)/ANO-RCC

N1	0.000000	0.000000	-0.025227
C2	0.000000	0.000000	1.478325
C3	0.000000	1.214300	2.146525
C4	0.000000	-1.214300	2.146525
C5	0.000000	1.206051	3.536580
C6	0.000000	-1.206051	3.536580
C7	0.000000	0.000000	4.230640
H8	0.000000	2.130879	1.596675
H9	0.000000	-2.130879	1.596675
H10	0.000000	2.135306	4.071056
H11	0.000000	-2.135306	4.071056
H12	0.000000	0.000000	5.302980
O13	0.000000	1.085870	-0.588645
O14	0.000000	-1.085870	-0.588645

Cartesian coordinates of nitrobenzene in Å. MP2(RHF)/def2-TZVPP

N1	0.000000	0.000000	2.528815
C2	0.000000	0.000000	1.056887
C3	0.000000	1.218070	0.390760
C4	0.000000	-1.218070	0.390760
C5	0.000000	1.207373	-1.000896
C6	0.000000	-1.207373	-1.000896
C7	0.000000	-0.000000	-1.697666
H8	0.000000	2.136888	0.956115
H9	0.000000	-2.136888	0.956115
H10	0.000000	2.144296	-1.539898
H11	0.000000	-2.144296	-1.539898
H12	0.000000	-0.000000	-2.778658
O13	0.000000	1.089782	3.094748
O14	0.000000	-1.089782	3.094748
